# Beyond the acceptance decision: integrating genetic findings into lifelong living donor care

**DOI:** 10.3389/ti.2026.17017

**Published:** 2026-08-06

**Authors:** Stefan Reuter, Daniela A. Braun, Klemens Budde, Barbara Suwelack

**Affiliations:** 1 Department of Medicine D, Transplant Nephrology, University Hospital Münster, Münster, Germany; 2 Safety of the Living Kidney Donor - German National Registry (SOLKID-GNR), Münster, Germany; 3 Department of Nephrology and Medical Intensive Care, Charité - Universitätsmedizin Berlin, Berlin, Germany

**Keywords:** donor follow-up, donor safety, genetic testing, kidney transplantation, living kidney donation, postdonation care, registry

Dear Editors,

The process of living kidney donation transforms a one-time acceptance or refusal decision into a lifelong relationship with an otherwise healthy volunteer. Genetic testing is often treated as similarly definitive, yet many results are variants of uncertain significance (VUS): findings for which evidence is insufficient to classify a variant as benign or pathogenic. Their meaning may change as population, segregation, functional, and genotype-phenotype data accumulate. In healthy donors facing a once-in-a-lifetime decision, variants initially considered insignificant may later become clinically relevant whereas likely pathogenic variants may remain clinically silent. Yet current care structures do not define who should monitor, reinterpret, and communicate updated genetic findings after nephrectomy. Targeted donor genetics must therefore be linked to registry-supported lifelong donor care.

In current practice, genetic testing in living donor evaluation is targeted: it is considered when family history, young donor age, genetically unexplained kidney disease in the intended recipient or family, or features such as persistent hematuria, albuminuria, cystic disease, recurrent nephrolithiasis, congenital urogenital anomalies, or extrarenal phenotypes suggest inherited risk, preferably after index-case testing. Universal testing would mean broad sequencing of all donor candidates irrespective of such suspicion. Schott et al. tested 46 approved donors prospectively and 122 donors who developed adverse renal outcomes retrospectively; pathogenic or likely pathogenic variants were identified in 4% and 19% of these cohorts, respectively, and VUS in 59% of all 168 donors [1]. These findings support targeted testing in high-risk constellations. However, the baseline prevalence of pathogenic variants in donors without adverse outcomes remains unknown, causality between identified variants and subsequent morbidity is uncertain, and many genetic results retain interpretive uncertainty. These data raise important questions about donor selection, counseling, and post-donation responsibilities.

In a dedicated renal genetics cohort, variant reclassification occurred in 5% of cases; when it did, it led to a new or revised diagnosis in 60% and significant management changes in 67%, with a median time to reclassification of 8.4 months [2]. Genetic results issued before donation cannot therefore be considered definitive. Baseline variant uncertainty combined with clinically meaningful post-issuance reclassification creates a longitudinal care obligation that current evaluation frameworks do not address.

Current practice resources guide testing indications, recommend index-case testing and counseling, and caution against inappropriate donor exclusion for VUS [3, 4]. KDIGO frames living donor evaluation and care as risk estimation, informed consent, and postdonation follow-up [5]. These documents define the predonation pathway, including use of genetics to inform donor selection, but do not assign responsibility for genetic findings afterward. Even in hereditary oncology, where variant reclassification is well recognized, it remains contested whether there is a duty to actively seek reclassification and, if a clinically relevant reclassification becomes known, who should recontact the patient [6]. Transplant medicine has no equivalent debate, let alone resolution, despite the additional ethical complexity of healthy-volunteer donors.

We propose integrating genetic findings obtained during donor evaluation into lifelong donor care across three dimensions. Clinical stewardship requires systematic documentation of genetic results and index-case diagnoses, a mechanism to receive clinically relevant updates from genetics or laboratory services, and cascade testing when index-case findings change. Longitudinal communication requires defined recontact pathways when relevant reclassifications are flagged or when a new genetic diagnosis is established in the family. Psychosocial support addresses anxiety or decisional burden from uncertain findings or late reclassifications. This approach does not treat every genetic uncertainty as a contraindication; it accepts that uncertainty must be managed after donation. Currently, no guideline designates whether anyone should actively seek reclassification, who should flag updates, who—the transplant centre, referring nephrologist, renal genetics service, or laboratory—should recontact donors, or whether donors themselves are expected to initiate follow-up after nephrectomy.

A positive or reclassified genetic finding rarely creates a genotype-specific therapy, but it should lower the monitoring threshold and narrow the time window for clinical detection. A donor carrying a heterozygous COL4A3 or COL4A4 variant or a COL4A5 variant may warrant earlier assessment for hematuria, albuminuria, and hypertension than one without known genetic risk—not because treatment differs, but because earlier detection enables earlier intervention. Health behavior and timely renoprotection may modify risk. As the DESCARTES working group emphasized, donor information, health-promoting behavior, and individualized long-term risk assessment are central after living donation [7]. Genetically informed postdonation surveillance should therefore address blood pressure, albuminuria, kidney function, lifestyle modification, and avoidance of nephrotoxins; renin-angiotensin-aldosterone system blockade and sodium-glucose cotransporter 2 inhibitor therapy should be initiated when guideline-supported indications arise [8].

The implementation question is where this longitudinal responsibility should reside. Routine clinical care is episodic and visit-dependent, not designed to track variant reinterpretation, family diagnoses, or psychosocial outcomes. The Safety of the Living Kidney Donor—German National Registry (SOLKID-GNR) offers a structural model, with sustained donor contact [9] and donor-reported outcomes that capture clinically relevant complications not reflected in centre records—an infrastructure also applicable to documenting genetic stewardship events such as relevant reclassifications, new family diagnoses, and psychosocial burden [10]. Embedding genetic findings into SOLKID-GNR would mean linking genetic results and index-case diagnoses to donor records, flagging relevant reclassification events and new family diagnoses, and collecting donor-reported psychosocial burden and clinical changes—without central genomic data storage. The registry would not itself reclassify variants; rather, it would provide a structure through which updates generated by genetics or laboratory services can be linked to donor follow-up. A national registry thus offers data infrastructure and a governance model: a defined institutional locus for genetic stewardship beyond donation.

Living kidney donation will always require balancing donor and recipient benefits through informed consent and shared decision-making. Targeted genetic testing in selected donors can improve this balance when it informs donor selection and the intensity of long-term postdonation care. Sequencing identifies what the genome contains; only structured, registry-supported lifelong follow-up determines what that information means for this donor—and who remains responsible for acting on it.
